# Phylogenetic and Expression Analysis of *CENH3* and *APOLLO* Genes in Sexual and Apomictic *Boechera* Species

**DOI:** 10.3390/plants11030387

**Published:** 2022-01-30

**Authors:** Evgeny Bakin, Fatih Sezer, Aslıhan Özbilen, Irem Kilic, Buket Uner, Mike Rayko, Kemal Melih Taskin, Vladimir Brukhin

**Affiliations:** 1 Bioinformatics Institute, 197342 Saint-Petersburg, Russia; eugene.bakin@gmail.com; 2 Department of Molecular Biology and Genetics, Çanakkale Onsekiz Mart University, Çanakkale 17100, Turkey; fatihsezer@comu.edu.tr (F.S.); buketuner@stu.comu.edu.tr (B.U.); 3 Department of Biology, Çanakkale Onsekiz Mart University, Çanakkale 17100, Turkey; aslihanozbilen@hotmail.com (A.Ö.); iremkilic72@gmail.com (I.K.); 4 Laboratory for Algorithmic Biology, Saint-Petersburg State University, 199004 Saint-Petersburg, Russia; mike.rayko@gmail.com; 5 Plant Genomics Lab, ChemBio Cluster, ITMO University, 191002 Saint-Petersburg, Russia; 6 Department of Plant Embryology and Reproductive Biology, Komarov Botanical Institute Russian Academy of Sciences, 197376 Saint-Petersburg, Russia

**Keywords:** *Boechera*, brassicaceae, *CENH3*, APOLLO, apomixis associated genes, cell division, apomeiosis

## Abstract

Apomictic plants (reproducing via asexual seeds), unlike sexual individuals, avoid meiosis and egg cell fertilization. Consequently, apomixis is very important for fixing maternal genotypes in the next plant generations. Despite the progress in the study of apomixis, molecular and genetic regulation of the latter remains poorly understood. So far *APOLLO* gene encoding aspartate glutamate aspartate aspartate histidine exonuclease is one of the very few described genes associated with apomixis in *Boechera* species. The centromere-specific histone H3 variant encoded by *CENH3* gene is essential for cell division. Mutations in *CENH3* disrupt chromosome segregation during mitosis and meiosis since the attachment of spindle microtubules to a mutated form of the *CENH3* histone fails. This paper presents in silico characteristic of *APOLLO* and *CENH3* genes, which may affect apomixis. Furthermore, we characterize the structure of *CENH3* by bioinformatic tools, study expression levels of *APOLLO* and *CENH3* transcripts by Real-Time Polymerase Chain Reaction RT-PCR in gynoecium/siliques of the natural diploid apomictic and sexual *Boechera* species at the stages of meiosis and before and after fertilization. While *CENH3* was a single copy gene in all *Boechera* species, the *APOLLO* gene have several polymorphic alleles associated with sexual and apomictic reproduction in the *Boechera* genera. Expression of the *APOLLO* apo-allele during meiosis was upregulated in gynoecium of apomict *B. divaricarpa* downregulating after meiosis until the 4th day after pollination (DAP). On the 5th DAP, expression in apomictic siliques increased again. In sexual *B. stricta* gynoecium and siliques *APOLLO* apo-allele did not express. Expression of the *APOLLO* sex-allele during and after meiosis in gynoecium of sexual plants was several times higher than that in apomictic gynoecium. However, after pollination the sex-allele was downregulated in sexual siliques to the level of apomicts and increased sharply on the 5th DAP, while in apomictic siliques it almost did not express. At the meiotic stage, the expression level of *CENH3* in the gynoecium of apomicts was two times lower than that of the sexual *Boechera*, decreasing in both species after meiosis and keep remaining very low in siliques of both species for several days after artificial pollination until the 4th DAP, when the expression level raised in sexual *B. stricta* siliques exceeding 5 times the level in apomictic *B. divaricarpa* siliques. We also discuss polymorphism and phylogeny of the *APOLLO* and *CENH3* genes. The results obtained may indicate to a role of the *CENH3* and *APOLLO* genes in the development of apomixis in species of the genus *Boechera*.

## 1. Introduction

Sexual reproduction is the main mode of reproduction of flowering plants. The major features of sexuality are meiosis and fertilization, the latter occurs through the fusion of haploid female and male gametes, giving rise to the formation of the genetically variable progeny. Genetic mutations and meiotic recombination provide permanent genetic changes, which are the source for evolution and adaptation of the population, as well as the basis for selection in agriculture. Despite the fact that sexual reproduction is energetically very expensive, the advantage of sex is that it forms a combination of useful mutations that are absent in asexual organisms [1,2]. Thus, the benefit of sexuality is that it helps to get rid of harmful mutations (Hill-Robertson effect) [3,4] and produces useful traits and variability. However, apart from the high energy costs, the disadvantage of sexual reproduction is the segregation of beneficial traits in subsequent generations, so that the offspring can lose useful combinations of their parental genes [5,6].

One of the mechanisms that can produce “clones” of mother plants is apomixis, which is a mode of asexual reproduction through seeds that has been identified in over four hundred of plant species [7,8]. In apomicts, meiosis and fertilization are modified or completely absent. Consequently, the embryo is formed without prior meiosis (by apomeiosis) and fertilization (i.e., by parthenogenesis), while endosperm development occurs either autonomously, i.e., without fertilization, or pseudogamously (by fertilization of the central cell) [6,8,9,10,11,12,13]. During double fertilization, intrinsic to all Angiosperms, the pollen tube transports two sperm cells (male gametes) to the embryo sac, one of which fertilizes the egg cell (female gamete) and the second one fertilizes the central cell, giving rise to a 2n embryo and a 3n endosperm, respectively. In sexual species, departures from the 2 maternal:1paternal (2m:1p) genome ratio in endosperm nuclei result in seed abortion. While in pseudogamous apomicts, endosperm ploidy varies according to the ploidy of the sperm and central cell. Deviations from 2m:1p genome ratio sometimes occur, demonstrating that the apomictic system is more resilient compared to sexual species [14].

Apomictic plants form genetically identical offspring. They dramatically influence the structure of the population playing important ecological role in the origin of polyploids and speciation [15,16]. Thereby these plants are excellent models for studying the mechanisms of the onset of meiosis and its replacement by apomeiosis, the formation of a seed in the absence of fertilization or under pseudogamy.

However, so far, little is known about the molecular background of apomixis and the genes associated with its triggering [17].

In this paper, we characterized the protein structure, evolution, and expression patterns of the *APOLLO* (for APomixis-Linked LOcus; encodes aspartate glutamate aspartate aspartate histidine exonuclease) gene, one of the important genes associated with apomixis in *Boechera* genus. It was shown that *APOLLO* have several polymorphic sex- and apo-alleles [18,19]. Apo-alleles of this gene are missing in sexual ovules and are up-regulated in apomeiotic ovules in *Boechera* plants. It was reported that genomes of apomictic plants are always heterozygous carrying at least one of the apo-alleles, while sexual genotypes were always homozygous for sex-alleles [18,19]. We showed that while sex-alleles were upregulated during meiosis in sexual plants and downregulated at the same stage in apomictic *Boechera* plants. Evolutionary *APOLLO* gene analysis presented in this study shows that sexual and apomictic species of *Boechera* are clustered in different clades in the phylogenetic tree based on the multiple protein sequence alignment. This could be partly because the *APOLLO* apo-alleles present in the genomes of apomictic species could acquire a new function [19]. Expression analysis of the *APOLLO* presented here showed that the expression levels of the gene dramatically decreased during meiosis in gynoecia of both apomictic and sexual species; however, after meiosis and fertilization, the expression of the *APOLLO* was upregulated in apomictic siliques compared to sexual ones.

We also characterize the *CENH3* gene, which is essential for cell division [20,21] and might affect the apomictic events. *CENH3* encodes the centromere-specific histone H3 variant. The accumulation of CENH3 provides an assembly site for a protein complex called kinetochore. The main function of the kinetochore is to bind chromosomes to spindle fibers during chromosome segregation in meiosis and mitosis [22]. CENH3 harbor two domains, a DNA-binding histone fold domain (HFD) and N-terminal tail domain. HFD is structurally similar to the same domain in H3 and is highly conserved across higher eukaryotes. However, the N-terminal tail of the CENH3 protein is highly variable even among closely related species and has meiosis-specific function [23]. Mutations at the N-terminal tail of *CENH3* disturb chromosome segregation in meiosis and often lead to sterility [20,24]. Null mutations in *CENH3* also cause chromosome elimination and were proposed as a tool to produce haploid plants [25,26,27,28].

In *Arabidopsis thaliana*, the *CENH3* mutants act like a haploid inducer parent when cross with a wild type plant [25,27] and the chromosomes in these mutants were lost in the developing embryo. The resulting plant contains a haploid set of the wild-type parent chromosomes, which can be induced to double its number. We investigated the structure and the expression profiles of *CENH3* in apomictic and sexual *Boechera* species (Brassicaceae) to further understand its role in meiotic chromosome segregation.

Plants from the *Boechera* genus are attractive models for research because both sexual and apomictic accessions are present within this genus. Moreover, plants from the *Boechera* genus are close relatives of *Arabidopsis thaliana*, which is very well studied in terms of molecular genetics and functional gene annotation [29,30,31]. In apomictic *Boechera* species, the *Taraxacum* type diplospory with pseudogamous endosperm development that requires fertilization of the central cell is reported [31,32,33,34]. Recently, the Hieracium type apospory and antennaria-type diplospory have also been reported in various Boecehra taxa [35]. Something worth noting is the apomictic accessions of *Boechera divaricarpa*, which is known as an interspecific hybrid between sexual species *B. stricta* and *B. retrofracta* or a closely related species [36]. Although diploid apomixis is an extremely rare condition in plants [37], both diploid and triploid apomictic *B. divaricarpa* lineages have been reported [31,38].

Our study revealed *CENH3* expression levels in the gynoecia/siliques during sexual and apomictic development. During meiosis and pollination *CENH3* expression demonstrated decreasing levels in both sexual and apomictic gynoecia and in the first day after pollination showed an increase in expression but in the gynoecia of sexual plants expression was almost two-fold higher than that in the gynoecia of apomicts. These results may indicate to a possible role that CENH3 might play in apomictic development in *Boechera* species.

## 2. Materials and Methods

### 2.1. Plant Material

The diploid sexual *B. stricta* (ES6, ID 500206; DG; DQ013050) and apomictic *B. divaricarpa* (ES9, 500209; BS) seeds were kindly obtained from Dr. Eric Schranz (Wageningen University & Research, Netherlands). *Boechera* plants were grown as described in [38,39]. Seeds of ES6 and ES9 accessions were germinated on moist filter paper after vernalization at 4 °C for 3 weeks in the dark to break seed dormancy. Then petri dishes containing the seeds were transferred into a growth chamber under the condition of 16 h light: 8 h dark (21 °C). Germinated seeds were transferred to a peat: perlite mix (1:4) for 4 weeks. Then the plants were grown at 10° C for 6 weeks. Flowering of the plants started in 6 weeks after the vernalization.

**Figure 1:**
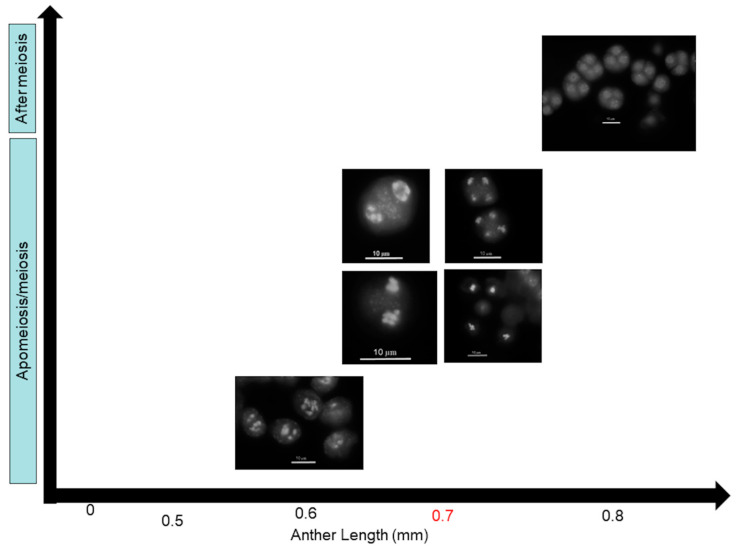
The male gametophyte development in diploid *Boechera* spp. Tissues for gene expression studies were collected as previously described [34,40].

Emasculation was performed on unopened flower buds by removing anthers with fine forceps. Hand pollination of stigmas was carried out with pollen from the same plant. Gynoecia/ siliques were collected one day before pollination (during meiosis according to gynoecium size, see [40]), right after pollination (after meiosis), on the 1st, 3rd, 4th and 5th days after pollination. Anthers were collected during and after meiosis (see Figure 1 [40]). The collected samples were immediately frozen in liquid nitrogen and stored at −80 °C until use.

### 2.2. APOLLO and CENH3 Genes Retrieval and Pre-Processing

Sequences of the *APOLLO* (Aspartate Glutamate Aspartate Aspartate histidine exonuclease) and *CENH3* (histone H3-like centromeric protein) genes in various *Boechera* species were obtained from the sources listed in Table 1 (also see in [31]). For *APOLLO* phylogenetic analysis, additionally 10 accessions from [18] listed in Table 2 (5 apo-alleles, 5 sex-alleles) were used.

For the species with available assembled and annotated genomes, a BLAST search for over-extracted proteins datasets was performed using *A. thaliana* peptide sequences as a query. For the other species (all *B. spatifolia* accessions from research [43] as well as *B. arcuata* and *B. divaricarpa* accessions) a local assembly using SRAssembler v1.0 was performed [44]. Consistency of the loci assembly results were checked with another approach, in which a whole-genome draft assembly was performed using Platanus software v1.2.1 [45] with a consequent genes search via Exonerate v2.2 run in protein2genome mode [46]. All the mentioned tools were run with default parameters.

The resulting parameters of the obtained assemblies are listed in Table S1 (Supplementary Materials). As one can see these assemblies have rather modest characteristics (N50 of about a few kilobases) as predicted earlier in [31]. Insufficient genome coverage in the datasets (30x in average), short reads length and high complexity of the genomes resulted in the fact, that most of the *B. spatifolia* accessions reported in [43] were excluded from the further analysis. In the excluded datasets, a significant divergence of the obtained sequences between two assembly methods and abnormally low their similarities with homologs in the other *Brassicaceae* species were detected. Thus, only Rosita3 accession was accepted for *CENH3* analysis. For *APOLLO* analysis Rosita3, Tiesiding2, Chicago2 and Royal2 accessions were accepted. In *B. puberula* and *B. perrenas* assemblies *APOLLO* gene was incomplete and thus these accessions were also excluded from the further gene analysis. It is worth noting that a short read length did not allow us to perform unambiguous diploid assemblies and distinguish different copies and alleles of the considered genes, potentially presenting in the genomes. Thus, all the downstream analysis was performed with consensus variants of the assembled genes. Sequences for non-*Boechera* species were retrieved directly from Phytozome v. 12.1 [47] and Uniprot [48] databases.

### 2.3. General Characteristics of CENH3 Gene

*CENH3* gene length and exon structure were directly extracted from both SRAssembler and Exonerate output. The molecular weight and isoelectric point of the proteins were calculated with Expasy’s ProtParam server [49]. Subcellular localizations of the *Boechera* proteins were predicted via CELLO v.2.5: subcellular localization predictor [50]. Conserved regions were investigated with CDD (Conserved Domain Database) [51]. The promoter regions were analyzed for regulatory motifs using PLANTCARE database [52].

### 2.4. Evolutionary Analysis of the CENH3 and APOLLO Genes

Multiple alignment of the obtained amino acid sequences was performed with MUSCLE algorithm [53] and then visualized with Jalview v2 [54]. The evolutionary history was inferred by the Maximum Likelihood method and w/freq. model [55]. As a result, the tree with the highest log likelihood was reconstructed. The percentage of trees in which the associated taxa clustered together is shown next to the branches (1000 iterations were performed). The tree was drawn to scale, with branch lengths measured in the number of substitutions per site. Evolutionary analyses were conducted in MEGA X [56]. Heatmap visualization was performed by R package Pheatmap [57].

### 2.5. CENH3 and APOLLO Genes Expression Studies

Total RNAs were isolated from gynoecium and anthers during meiosis and siliques 1–5 days after pollination (DAP) according to [40] with Purelink RNA mini kit (Invıtrogen–12183018A) and TRIzol reagent (Invitrogen, 15596018). The RNA quality was checked on 1% agarose gel electrophoresis, the concentration of RNA was measured with a Qubit 2.0 Fluorometer (Life Technologies, Carlsbad, CA, USA). Before the cDNA synthesis, 1 µg of total RNA was treated with Rnase-free Dnase I (Thermo Scientific, EN0521) for 30 min at 37 °C. Reverse transcription reactions were performed using 1 µg of total RNA and High-Capacity cDNA Reverse Transcription Kit (4368814; Applied Biosystems, Foster City, CA, USA) according to the manufacturer’s instructions. Expression of the *CENH3* and *APOLLO* genes were analyzed with the 7500 real-time PCR system (Applied Biosystems) according to the manufacturer’s instructions. The efficiency of the primers used in the study was evaluated with the standard curve experiments, only the primers with 90–100% efficiency were used. The UBQ (Polyubiquitin) gene was used as the endogenous control. No template (water instead of the template) and RT (water instead of reverse transcriptase enzyme in cDNA synthesis) controls showed negative amplification. Primers sequences were BOECHUBQ_F: 5′-GGCTAAGATCCAGGACAAGGAAGGTAT-3′, BOECHUBQ_R: 5′-CTGGATGTTATAGTCAGCCAAAGTGCG-3′ for UBQ amplification; APOLLO_F: 5′-CGGAGTTCTCTCTGCACCTAC 3′ and APOLLO_R: 5′-TTCGTCCGTGGAGAATGTCG-3′ for APOLLO analysis; Cenh3_F 5′-CAACTCCTACAACTTCACCAGCTACTG-3′ and Cenh3_R 5′-TTGTGAACCTTGTGGCCTAGCATATC-3′ for CENH3 analysis. All reactions were performed in three biological and three technical replicates. Reactions included 10 µL SYBR Green PCR Master Mix (Applied Biosystems–4367659), 0.6 µL primers (10 µM stock), 1 µL cDNA template (50 ng/µL), 7.8 nuclease-free water. The PCR cycling conditions were as follows: pre-denaturation at 95 °C for 10 min, denaturation at 95 °C for 15 s, annealing at 60 °C for 30 s, and elongation at 72 °C for 60 s for 40 cycles. The data were analyzed with expression suite software (Applied Biosystems) with the ∆∆Ct method.

## 3. Results

### 3.1. Characteristics of CENH3 Gene and Protein Isoforms

**Figure 2:**
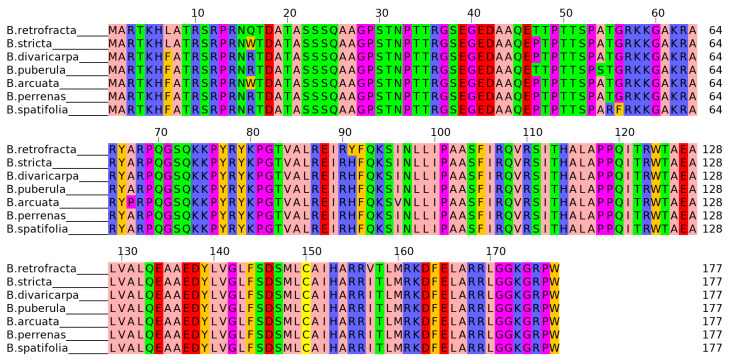
Multiple alignment of CENH3 proteins in the considered *Boechera* sp. Multiple alignment was performed using MUSCLE software and visualized in Jalview.

General characteristics of the *CENH3* gene and CENH3 protein in seven studied *Boechera* species is given in Table 3. In all these species CENH3 protein isoforms showed high similarity to each other with similarity index not less than 97% (at both the nucleotide and protein levels). All *Boechera* CENH3 protein sequences contained a Histone H3/CENP-A (IPR000164) conserved domain, were 177 a.a. long and predicted to be localized in the nucleus. CENH3 protein alignment results are represented in Figure 2.

Among the seven investigated *Boechera* species 10 polymorphic amino acid positions in CENH3 protein were detected. *B. stricta* and *B. divaricarpa*, which were further subjected to the comparative expression analysis of *CENH3* gene, had only two polymorphic amino acids in N-tail positions 7 and 16, which are located out of the Histone H3 conserved domain.

**Figure 3:**
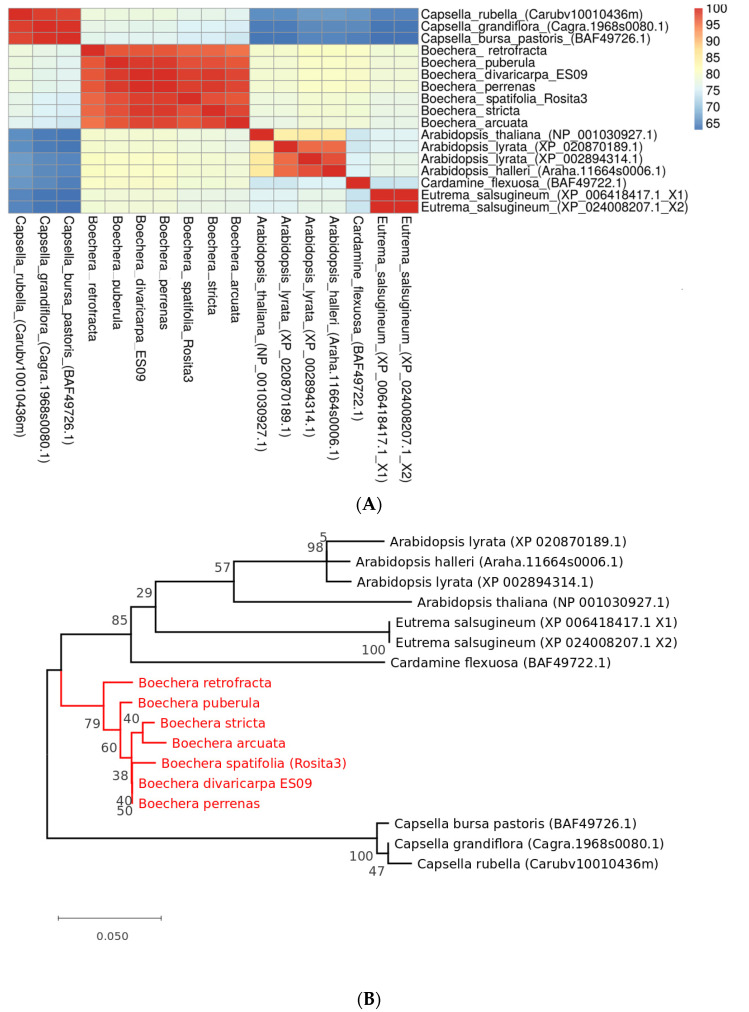
(**A**) Similarity matrix and (**B**) Phylogenetic tree of CENH3 (histone H3-like centromeric protein) in seven species of interest. Sequences of *Capsella* species were used as an outgroup. All *Boechera* species are grouped in one clade. Numbers near nodes represent corresponding bootstrap support. Phylogenetic tree was reconstructed using Maximum Likelihood method. The division value is an average number of substitutions per position.

The CENH3 similarity heatmap matrix and reconstructed phylogenetic tree are shown in Figure 3A,B, respectively. The heatmap demonstrates that CENH3s in all *Boechera* species are almost identical, which is consistent with the protein alignment analysis (Figure 2) and are very similar (≥80%) to *Arabidopsis* and most of the other representatives of Brassicaceae studied. The *CENH3* gene is represented by only one copy per genome of each investigated species. The evolutionary history was estimated with the help of the highest log likelihood. The reconstructed phylogenetic tree (Figure 3B) was rooted with *Capsella* spp. As an out-group. All *Boechera* species were grouped in one clade, which was separated from *Arabidopsis* spp., *Eutrema* spp., *Cardamine* sp., suggesting that unlike APOLLO, (see Section 3.5) CENH3 protein sequences do not reflect the mode of propagation within the *Boechera* spp.

### 3.2. CENH3 Promoter Characteristic

**Figure 4:**
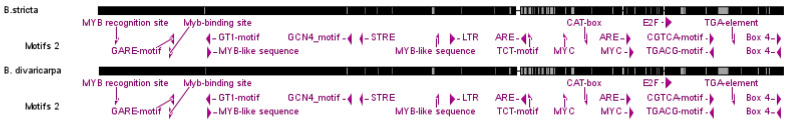
Motifs found in 1000 bp upstream of the transcription start site of *BsCENH3* and *BdCENH3*.

Since it was found that genomes of all studied species contain only a single copy of the *CENH3* gene, we decided to investigate and compare the structure of the *CENH3* gene promoters in two species, sexual *B. stricta* and apomictic *B. divaricarpa*. A total of 1000 bp upstream regions from the first codon (ATG) of *BsCENH3* and *BdCENH3* genes were analyzed. Several regulatory elements and core promoter regions like TATA and CAAT-box within both *CENH3* promoters were found. Among the regulatory motifs, MYB transcription factors recognition and binding sites were common for both *CENH3* promoters. MYB transcription factors are encoded by several genes that control plant development, differentiation, stress resistance, and defense [58]. The presence of several MYB binding motifs might indicate to a role of MYB transcription factors in *CENH3* regulation. E2F transcription factor binding sites at −163 positions of *BsCENH3* and *BdCENH3* were found as well. At −115 site *BdCENH3* and *BsCENH3* had a single polymorphic site (GCGGGAAA/ GCGGGAAG). Promoter regions also included several light response motifs like Box 4, GT1-motif and TCT-motif. Hormone response elements like gibberellin responsive element (GARE-motif) and auxin-responsive elements (TGA-element) were detected in the *CENH3* promoters of both species. A cis-acting regulatory element involved in the MeJA-responsiveness (CGTCA-motif), cis-acting element involved in low-temperature responsiveness (LTR), cis-acting regulatory element involved in circadian control, cis-acting regulatory element related to meristem expression (CAT-box) and cis-regulatory element involved in endosperm expression (GCN4_motif) were also found in the *CENH3* promoter (Figure 4). Our research of promoter regions of *BsCENH3* and *BdCENH3* genes did not reveal significant differences (Figure 4). Therefore, we decided to check the expression levels of *BsCENH3* and *BdCENH3* genes in the meiotic gynoecia and young siliques just after fertilization.

### 3.3. CENH3 Expression during and after Meiosis in Gynoecia/Siliques and Anthers

**Figure 5:**
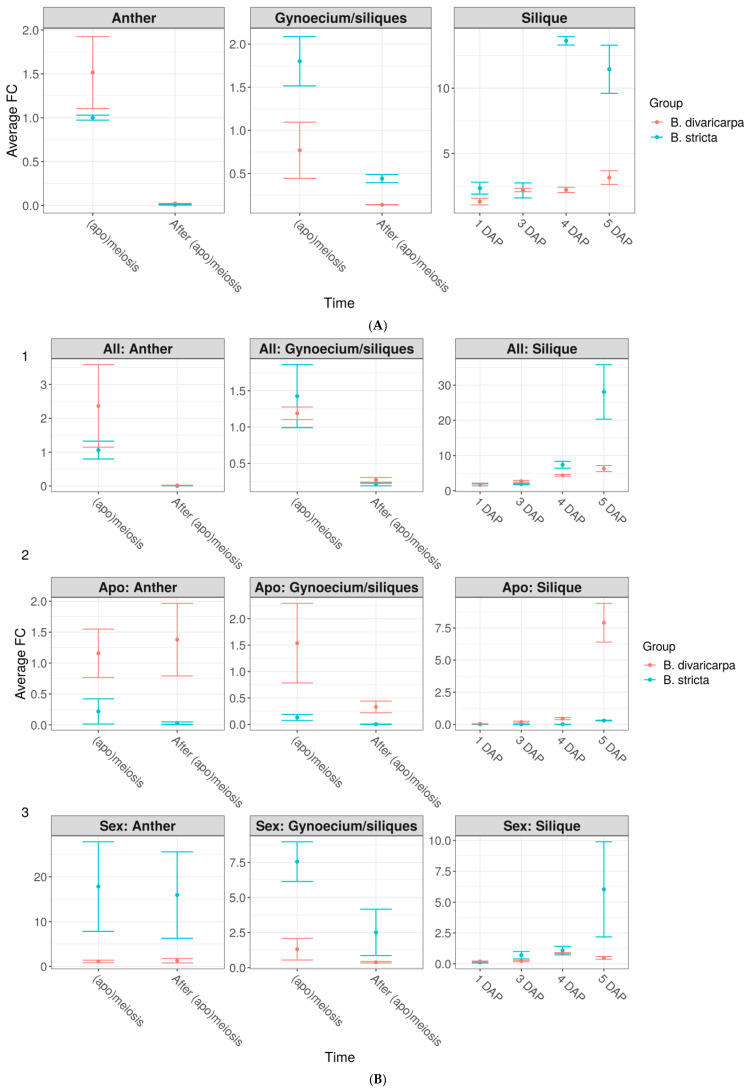
Expression levels of *CENH3* and *APOLLO* genes in diploid apomict and sexual *Boechera* plants: *B. divaricarpa* and *B. stricta* in (**A**) Expression patterns of *CENH3*; (**B**) Expression patterns of *APOLLO:* (1) Expression of *APOLLO* using universal primers for both sex- and apo-alleles. (2) Expression of *APOLLO* apo-alleles in anthers, gynoecium, and siliques of apomictic *B. divaricarpa* and sexual *B. stricta* plants. (3) Expression of *APOLLO* sex-alleles in anthers, gynoecium, and siliques of apomictic *B. divaricarpa* and sexual *B. stricta* plants. Bars indicate ± standard errors of fold change levels. Days After Pollination (DAP).

Comparative expression analysis of *CENH3* gene was performed in gynoecia and anthers from unopened flower buds (before pollination) and in siliques 1–5 DAP (Figure 5A; Table S2 in Supplementary Materials) to cover developmental stages around meiosis, gametophyte development, and seed set in diploid apomict *B. divaricarpa* and diploid sexual *B. stricta*. For investigation after the pollination, only the extending siliques were used to make sure that the pollination had occurred and seed development had started. It was found that, during meiosis/apomeiosis, the *CENH3* gene is intensely expressed in gynoecium of both sexual *B. stricta* and apomictic *B. divaricarpa,* over two folds higher expression level was observed in *B. stricta. CENH3* was significantly downregulated after meiosis, yet the expression level in *B. stricta* was higher than that in *B. divaricarpa* (Figure 5A). During three days after pollination, the level of *CENH3* expression in siliques remained low in both studied species. On the 4th DAP, the expression of *CENH3* significantly increased in siliques of *B. stricta* and was more than 6 times higher than that in *B. divaricarpa,* while in the latter it remained practically unchanged. The same trend persisted in siliques of both species on the 5th DAP; however, the difference between sexual and apomictic species was less and differed by 3.7 times (Figure 5A).

We also investigated the expression levels of the *CENH3* gene in *B. stricta* and *B. divaricarpa* anthers around meiosis. During meiosis in anther tissues the level of the *CENH3* expression in *B. stricta* was 1.5 times lower than in *B. divaricarpa*. By the end of meiosis, expression of *CENH3* was down regulated almost completely in anthers of both species (Figure 5A).

The observed difference in the level of *CENH3* gene expression between sexual and apomictic accessions could be either due to differences in regulation of expression by the transcription factors and genes responsible for their expression or due to epigenetic factors that might regulate the expression at a post-translational level. A decreased level of expression of CENH3 during and after meiosis in the generative organs of apomictic *B. divaricarpa* compared with sexual *B. stricta* may be one of the reasons of the meiosis disturbance in apomicts.

### 3.4. APOLLO Expression during and after Meiosis in Gynoecia/Siliques and Anthers

Along with the analysis of the *CENH3* gene dynamics, the expression of the *APOLLO* gene apo- and sex- alleles were studied during and after meiosis in gynoecium and anthers and after pollinations in siliques of sexual diploid *B. stricta* and apomictic diploid *B. divaricarpa* 1–5 DAP (Figure 5B; Table S2 in Supplementary Materials). Since *B. stricta* has no presence of apo-allele in the genome, we used it as a negative control.

In apomeiotic gynoecium of *B. divaricarpa* the level of *APOLLO* apo-allele expression was four times higher compared to its expression after meiosis. During four DAP, apo-allele expression was downregulated in siliques of *B. divaricarpa* almost to zero; however, on the 5th DAP in *B. divaricarpa* siliques, the expression of the *APOLLO* apo-allele sharply increased, while in *B. stricta,* as expected, the expression was not observed. (Figure 5B). Regarding the expression of sex-allele, the opposite was found. During meiosis gynoecium of apomictic *B. divaricarpa* demonstrated six times lower expression of the *APOLLO* sex-allele compared to the sexual *B. stricta.* Whereas after meiosis, the expression of the *APOLLO* sex-allele was strongly downregulated in gynoecium of both species, nevertheless, the expression level in *B. stricta* remained six times higher than that in *B. divaricarpa*. After pollination, the expression of the sex-allele almost completely declined in siliques of both species, and then from the 3 DAP, started gradually increasing to a sharp rise in the expression on the 5th DAP, while in siliques of sexual *B. stricta*, the expression was higher than that in apomicts of *B. divaricarpa* in 12 times.

The expression levels of the *APOLLO* apo-allele in meiotic anthers of *B. divaricarpa* were slightly upregulated after meiosis, whereas in the anthers of sexual *B. stricta* there was no expression as it should be (Figure 5B). As for the *APOLLO* sex allele expression in the anthers, its level remained rather high during and after meiosis in the sexual *B. stricta* and was very low during and after meiosis in anthers of apomictic *B. divaricarpa* (Figure 5B).

Using universal primers to detect the expression of both sex- and apo-alleles, we found that in anthers during meiosis, the total expression of *APOLLO* alleles was high in sexual and apomictic species. It was strongly downregulated in the anthers of both species soon after the end of meiosis (Figure 5B). As for the expression of the *APOLLO* alleles in gynoecium and siliques of both species, noticeable differences between sexual *B. stricta* and apomictic *B. divaricarpa* began to be observed only by the 5th day after pollination. Namely, during meiosis in gynoecium of both species, the expression of *APOLLO* was approximately the same, which was strongly downregulated after meiosis. After pollination the expression levels remained low up to the 4th DAP, then the level of gene expression in siliques of both species began to increase on the 5th DAP. In this stage, the expression of *APOLLO* remained still low for the apomicts, while for the sexual plant it jumped almost 5 times up (Figure 5B).

Thus, for the first time, we distinguished between the expression levels of apo- and sex-alleles of *APOLLO* in the generative organs of two *Boechera* species, differing in the mode of reproduction. Our studies have shown that the *APOLLO* apo-allele is upregulated in gynoecium and anthers during meiosis and in siliques on the fifth DAP in apomicts, while this allele is not expressed in sexual plants. The sex-allele showed the opposite picture: in sexual *B. stricta*, it was upregulated in the gynoecium and anthers during meiosis and on the fifth DAP, while the expression of this allele in *B. divaricarpa* remained at a very low-level during/after meiosis in anthers and the first days after pollination. Revealed interesting patterns of *APOLLO* expression were somewhat different from those previously published [18], the difference observed in *APOLLO* expression in our study, may also be tissue and species specific.

### 3.5. Phylogenetic Analysis of APOLLO Gene

**Figure 6:**
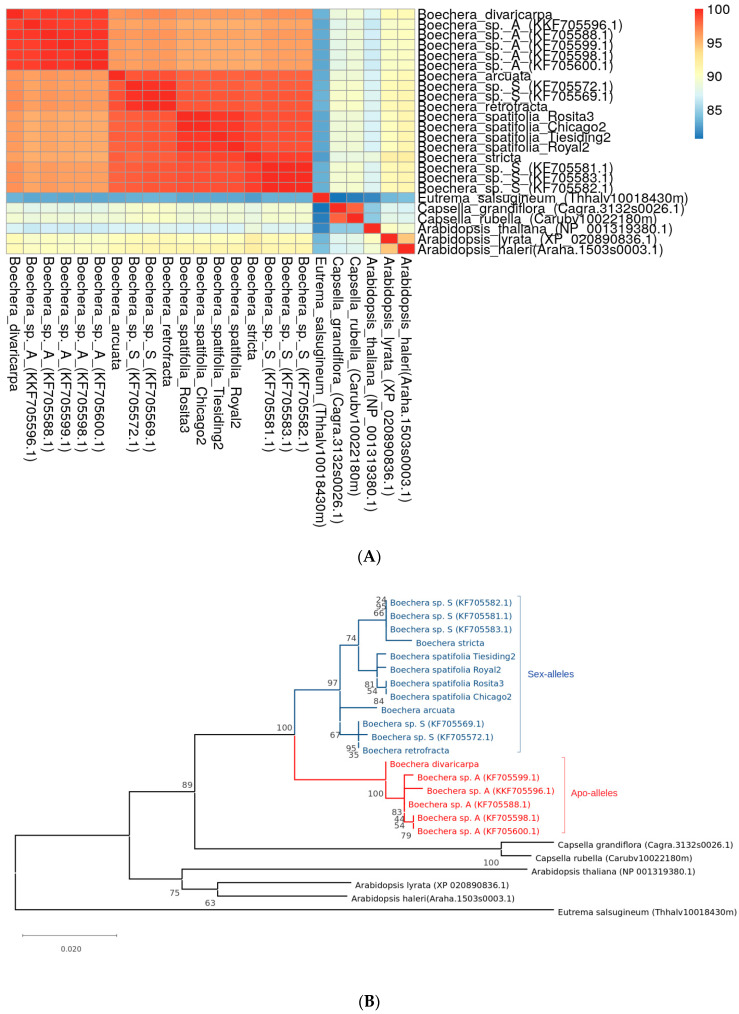
Similarity matrix (**A**) and Phylogenetic tree (**B**) of *APOLLO* in species of interest. Numbers near nodes represent corresponding bootstrap support. Phylogenetic tree was reconstructed using Maximum Likelihood method. The *Boechera* species are clustered in two separate subclades according to the mode of reproduction: the first subclade contains the homozygous accessions for sex-alleles and the second contains accessions with apo-alleles. The division value is an average number of substitutions per position.

A preliminary analysis of the *APOLLO* gene and protein orthologs was reported previously [19]; therefore, the objective of this research was to study the phylogeny of *APOLLO* gene using additional available in open access sequenced genomes of *Boechera* species with sexual and apomictic mode of reproduction by translating *APOLLO* nucleotide sequences into proteins and apply multiple alignment method for obtaining amino acid sequences using MUSCLE software and the Maximum Likelihood method to construct a phylogenetic tree. The heatmap shown in Figure 6A demonstrates that all compared APOLLO proteins of *Boechera* are rather similar (≥92.5%) despite the presence of five apo-alleles and five sex-alleles of that gene (see Table 2 in Methods). Phylogenetic tree of *APOLLO* (Figure 6B) shows that the *Boechera* species are clustered in two separate subclades according to the mode of reproduction, i.e., branches in the tree were grouped by genes rather than by species: the first subclade contains the homozygous for sex-alleles accessions reproducing sexually and the second contains accessions with apo-alleles that reproduce via apomixis. At the same time, all *Boechera* species represented in the phylogenetic tree are clustered in a common clade, separating them from the other genera of the Brassicaceae family (similarity ≥ 82.5%).

## 4. Discussion

Apomixis via clonal seeds produces the offspring, that are genetically identical to the maternal plant. Understanding of the genetic components that regulate apomixis is very important for studying plant development and evolution, in addition, the introduction of apomixis in agricultural plants would allow a long-term fixation of complex genotypes, including F1 hybrids, often used in agriculture. However, the molecular mechanisms underlying apomixis are poorly understood. Namely, the factors inducing avoidance or modification of meiosis (apomeiosis) and parthenogenesis. To study the genes regulating meiosis and embryogenesis in comparison of sexual vs. apomictic plants we use a convenient model plants from the *Boechera* genus, that comprise species naturally reproducing both by sexual and apomictic ways and show features of hybrid origin [31].

In this paper we studied similarity of homologs and phylogeny of *CENH3* and *APOLLO* genes that might affect some components of apomixis and compared the expression patterns of these genes in apomictic and sexual plants of *Boechera*.

The *CENH3* gene plays an important role in cell divisions and genome elimination when mutated. Mutations in *CENH3* of *Arabidopsis thaliana* cause disturbed meiotic chromosome segregation [20,21] that was also used to induce genome elimination in *A. thaliana* and rice. In hybrids of *cenh3* mutant lines with diploid wild type plants the *cenh3* line genome was eliminated [59]. We showed that *CENH3* is a single-copy gene and that its structure is almost identical among the seven studied *Boechera* species irrespective of the reproduction (sexual or apomictic) mode. Polymorphic sites were mostly found at the N-tail protein regions, although *B. retrofracta* and *B. arcuata* had one site at 91 a.a. and two polymorphic sites at 67 a.a. and at 96 a.a. respectively, in conservative Histone H3/CENP-A domain. Variability within the N-terminal tail might lead to apomeiosis, since it influences the chromosome segregation in meiosis [20,24], although this assumption still needs to be tested on mutant lines with the replacement of the corresponding polymorphic sites. Still the similarity index between all studied *Boechera CENH3* was ≥97% (both at nucleotide and protein levels). Furthermore, in the *CENH3* phylogenetic tree, performed by multiple alignment, all studied *Boechera* species were clustered into the same clade, although being very close to other Brassicaceae species from *Arabidopsis*, *Eutrema*, *Cardamine* genera. The *CENH3* expression profile analysis showed that during meiosis expression levels of *BsCENH3* in gynoecium of sexual *B. stricta* was more than twice as high compared with *BdCENH3* in gynoecium of apomictic *B. divaricarpa.* By the meiosis time, expression levels of both these genes dramatically dropped in gynoecia. After pollination, the expression *CENH3* significantly increased in *B. stricta* siliques by 4th DAP still remaining low in siliques of *B. divaricarpa*. The lower expression levels before and after pollination in *B. divaricarpa* (ES9) could indicate a feasible *CENH3* role in apomeiosis and initiation of parthenogenesis. In the meiotic anthers of *B. divaricarpa*, the expression of *CENH3* was 1.5 times higher than that in *B. stricta*, which may be associated with apomeiosis during pollen maturation. After meiosis, the level of gene expression dropped to zero in the anthers of both species.

In sexually reproduced plants, two sperms enter the embryo sac, while one of them fuses with the central cell nucleus that further forms endosperm, the second fertilizes the egg, in contrast, during pseudogamous apomixis, the embryo develops without fertilization, which is the cause of the “spare sperm problem” [60,61]. In apomicts fertilization of the central cell with haploid sperm generally leads to a 4m:1p genomes ratio in endosperm cells, which causes seed abortion [62]. Apomictic species can tolerate such deviations in endosperm via changing their imprinting systems; however, preventing the ‘spare sperm’ from fusing with the central cell nucleus might also be important [63]. The fusion of reduced or unreduced ‘spare sperm’ to the central cell can potentially affect the parental genomic ratio. Therefore, it is not known how the central cell in apomicts can avoid fertilization by enhancing a polyspermy barrier for that ‘spare sperm problem’ [61]. Thus, different expression behavior of *CENH3* before and after pollination in *B. divaricarpa* vs. *B. stricta* gynoecium/silique might suggest its involvement in apomictic development such as elimination of the chromosomes from male gamete during endosperm development. However, this assumption requires the further proof.

Promoter analysis revealed the presence of several MYB transcription factor binding and recognition motifs within the promoter regions of *BsCENH3* and *BdCENH3*. These motifs in *Boechera CENH3* promoters might suggest a regulation of these genes by a MYB family proteins. A study on *Arabidopsis CENH3* promoter region and its regulation revealed two E2F binding region at −163 and −115 sites [62]. Our analysis of these regions in *Boechera* species detected the motif at −163 site in *BsCENH3* and *BdCENH3*. At −115 site *BdCENH3* and *BsCENH3* had a single polymorphic site (GCGGGAAA/GCGGGAAG). Further functional studies on the *Boechera CENH3* gene and its epigenetic and transcription regulation could elucidate a functional difference of *CENH3* between apomictic and sexual *Boechera*.

Concerning *APOLLO,* it is the only so far found gene comprising the alleles with apomixis-associated polymorphism in *Boechera* species [18]. Thus, identification of *APOLLO* apo-alleles might be used as molecular markers to spot apomictic individuals among the *Boechera* species. Earlier it was shown that *APOLLO* encodes the exonuclease NEN3 and suggested an evolutionary scenario where after series of duplications one of the NEN3 protein copies of the *Boechera* ancestors acquired an alter function leading to apomictic development from the sexual state [19]. Moreover, it was demonstrated that apo-alleles are under a positive selection [19]. In the current study, we retrieved five apo- and five sex-alleles from [18] to screen the genomes of ten *Boechera* species with different reproduction modes as well as other Brassicaceae species from the *Arabidopsis*, *Eutrema*, *Cardamine*, and *Capsella* genera to perform *APOLLO* gene phylogenetic analysis. The results showed that sexual and apomictic *Boechera* species are clustered into the separate sub-clades, while being very similar to each other and to other Brassicaceae species. As well as for the *CENH3* gene, functional studies of the *APOLLO* alleles and their putative epigenetic and transcriptional regulation in *Boechera* species is required in order to find out if there are functional differences between sexual and apomictic accessions. However, the latter will only be possible when good quality diploid genome assemblies of the studied species have been implemented. Investigation of *APOLLO* expression in pre- and after meiotic gynoecium and anthers and after meiosis in siliques of sexual diploid *B. stricta* and apomictic diploid *B. divaricarpa* 1–5 DAP have been performed. The upregulation of the *APOLLO* apo-allele in *B. divaricarpa* apomicts after meiosis in the anthers and gynoecium and the decrease in the level of its expression with the onset of embryo sac formation may indicate to a certain relationship between the expression of the *APOLLO* apo-allele in apomicts and apomeiosis. This is consistent with the previously published data that the *APOLLO* apo-allele is exclusively expressed in ovules of apomictic *Boechera* species around stage of meiosis [18]. The upregulation of the *APOLLO* sex-allele in anthers and gynoecium of sexual *B. stricta* during and after meiosis and very low levels of its expression in apomicts is possibly associated with a connection of the sexual allele of the Aspartate Glutamate Aspartate Aspartate histidine exonuclease gene (*APOLLO*) with meiosis and further postmeiotic processes. However, the increase in the expression of apo- and sex-allele in siliques on the 5th DAP in apomicts and sexual species respectively, requires further explanation. Upregulation of *APOLLO* apo- and sex-alleles in meiotic anthers in apomictic *B. divaricarpa* two folds higher than in sexual *B. stricta* may indicate to a role of *APOLLO* in apomeiosis or meiosis during pollen formation; moreover, the male gametophyte development in apomictic *B. divaricarpa* were reported to produce both reduced and nonreduced gametes [38,39]. Therefore, we infer that *APOLLO* may have species specific function for the regulation of meiosis/apomeiosis in *Boechera* and have diverse functions in pseudogamous apomicts compared to sexual relatives.

In conclusion, a detailed knowledge of the structure, phylogeny of genes related to apomixis and the dynamics of their expression can presumably help to better understand the nature and regulation of apomixis vs sexual reproduction and facilitate further study of the evolutionary, ecological, and population role of apomixis. However, for more accurate studies on the phylogeny and evolution of the *Boechera* species, it is necessary to have a good quality diploid level whole genome assembly for these species, which we are now actively working on.

## Figures and Tables

**Table 1:** Genome sequencing data for *Boechera* genus.

Species	Reproduction Mode	Raw Data NCBI Accession	Genome Assembly	Genome Annotation	Reference
*B. stricta*	Sexual	SRR396760 SRR396762 SRR396756	Yes	Yes	[41]
*B. retrofracta*	Sexual	SRR3929707	Yes	Yes	[19]
*B. puberula*	Sexual	ERX2578777 ERX2578776	Yes	No	[42]
*B. spatifolia* (Rosita3)	Sexual	SRR5116719	No	No	[43]
*B. spatifolia* (Tiesiding2)	Sexual	SRR5116723	No	No	[43]
*B. spatifolia* (Chicago2)	Sexual	SRR5116732	No	No	[43]
*B. spatifolia* (Royal2)	Sexual	SRR5116730	No	No	[43]
*B. arcuata*	Sexual	SRR6448790	No	No	n/a
*B. divaricarpa*	Apomictic	SRR3500627 SRR3500628	No	No	n/a
*B. perennas*	Apomictic	SRR6448882	No	No	n/a

**Table 2:** *Boechera* accessions used for investigation of *APOLLO* gene (from [18]).

GenBank Accession	Sample ID	Allele Type
KF705583.1	369S2_S3	Sex-allele
KF705582.1	376S2_S5	Sex-allele
KF705581.1	355S2_S3	Sex-allele
KF705569.1	329S2_S1	Sex-allele
KF705572.1	385S2_S11	Sex-allele
KF705596.1	43A3_A3	Apo-allele
KF705598.1	1A2_A6	Apo-allele
KF705600.1	11A2_A1	Apo-allele
KF705599.1	11A2_A3	Apo-allele
KF705588.1	33A2_A5	Apo-allele

**Table 3:** General characteristics of *CENH3* gene and CENH3 protein in studied seven *Boechera* species.

Parameter	Value
Gene length	2231–2298 b.p.
Number of exons	10
Protein length	177 a.a.
Molecular weight	19,616.82 ± 60.4
Theoretical pI	11.25 ± 0.10
Subcellular Localization	Nuclear
Conserved Domains	Histone H3/CENP-A (from 57 a.a. to 172 a.a.)

## Data Availability

Data available in a publicly accessible repository. The data presented in this study are openly available via refs [18,31,34,40,43], see Table 1 and Table 2 and Figure 1 of the current paper. All information on the initial data is mentioned in the Materials and Methods paragraph. The data presented in this study are available in Supplementary Materials.

## Supplementary Materials

The following supporting information can be downloaded at: https://www.mdpi.com/article/10.3390/plants11030387/s1, Table S1—List of assemblies’ characteristics, Table S2—Expression levels of Apomictic, sexual, and both alleles of APOLLO gene and CENH3 gene.
